# Faster rates of post-puberty kidney deterioration in males is correlated with elevated oxidative stress in males vs females at early puberty

**DOI:** 10.1186/1471-2164-8-221

**Published:** 2007-07-09

**Authors:** Li Li, Susanne N Boehn, Xiaolei Yu, Qingqin Zhang, Marc Kenzelmann, Dieter Techel, Salah A Mohamed, Petra Jakob, Bettina Kraenzlin, Sigrid Hoffmann, Norbert Gretz

**Affiliations:** 1 Medical Research Center, University of Heidelberg, Theodor-Kutzer-Ufer, 68167 Mannheim, Germany; 2 The First Affiliated Hospital, Xinxiang Medical College, 453003 Xinxiang, China; 3 Department of Cellular and Molecular Biology, German Cancer Research Center, Im Neuenheimer Feld 280, 69120 Heidelberg, Germany; 4 Klinikum Bremen-Mitte, 28177 Bremen, Germany; 5 University Clinic SH-Campus Luebeck, Department of Cardiac Surgery, Ratzeburger Allee 160, 23538 Luebeck, Germany

## Abstract

**Background:**

Post-puberty deterioration of kidneys is more rapid in males than in females. To reveal the underlying molecular mechanisms for this difference, we analyzed gender-dependent gene expression in kidneys of three groups of 36 day-old rats.

**Results:**

The number of genes exhibiting gender-dependent expression was highly influenced by the genetic background of the rat group examined. 373, 288 and 79 genes showed differential gene expression between males and females (p = 0.001) in US, Mhm and Mhm*BN rats, respectively. Of all gender dependently expressed genes, only 39 genes were differentially expressed in all tested groups and the direction of expression change was the same for those genes for all groups. The gene expression profile suggests higher metabolic and transport activities, enhanced cell proliferation, elevated oxidative stress, and altered vascular biology in males. Furthermore, elevated levels of superoxide anion (two- to three-fold) in males compared to females were detected at early puberty, but neither at pre-puberty nor at late puberty/early adulthood.

**Conclusion:**

Our data suggest that early puberty, with gender-related elevation in oxidative stress in males, is a key compromising factor on kidneys in males.

## Background

Post-puberty deterioration and aging of kidneys seem to occur more rapidly in males than in females, characterized by shorter telomeres in males [1,2]. It is also well documented that many kidney diseases, such as hypertensive and diabetic renal diseases, glomerulonephritis, and polycystic kidney disease (PKD), have an earlier onset, higher prevalence and more rapid progression in males compared with females in human, mouse and rat [3-6].

Existing data support strong influences of sex hormones on renal function [6-8]. Gender differences in kidney function emerge at puberty, when sex hormones appear, and diminish later in life when females enter menopause. This difference can be greatly reduced or eliminated by orchiectomy, ovariectomy or hormone blockage [5,6]. Estrogen has been reported to protect against kidney deterioration, probably via its anti-oxidative and anti-inflammatory effects [7]. Androgens, on the other hand, have been shown to enhance fibrogenesis [9]. The exact mechanisms by which sexual hormones trigger the gender difference in kidney function are not yet well understood.

To reveal the underlying molecular mechanisms for the difference between genders in kidney function, the gender-dependent gene expression in kidneys of three groups of 36 day-old rats was evaluated with microarrays.

The influence of the genetic background of experimental rats on the gender-dependent gene expression is not yet well documented. Therefore, to define a list of genes with general gender-dependent gene expression, transcriptional analysis was performed in three rat groups in parallel.

Transcriptional analysis revealed the gender-dependent expression for genes of five different functional groups and suggested the elevated oxidative stress in males compared with females. Furthermore chemiluminescence measurements recorded elevated oxidative stress at early puberty, but neither at pre-puberty nor at late puberty/early adulthood. Our data suggest that early puberty, with gender-related elevation in oxidative stress in males, is a key compromising factor on kidneys in males.

## Results

### Gender-dependent gene expression was highly dependent on the genetic background of rat groups

**Figure 1:**
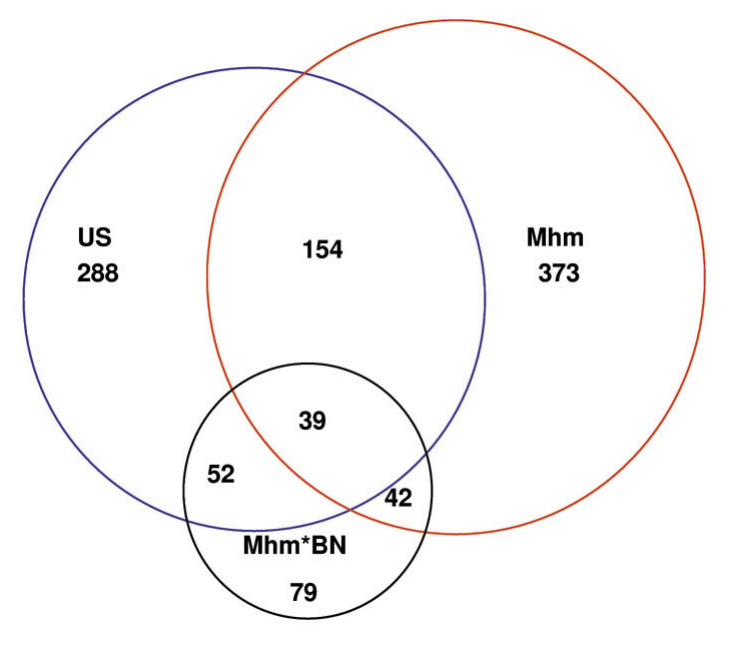
**The number of genes differentially expressed by gender was strongly influenced by genetic background**. Gender-dependent gene expression was examined in kidneys of 36 day-old US, Mhm, and Mhm*BN rats with Affymetrix arrays Rae230A. In this Venn diagram the number of genes with gender-dependent expression (p = 0.001) in the respective groups is depicted.

Gender-dependent gene expression in kidneys of three groups of 36 day-old rats was evaluated using microarrays. The number of genes exhibiting gender-dependent expression turned out to be highly influenced by the genetic background of the rat group examined (figure 1). In US, Mhm, and Mhm*BN rats, 373, 288 and 79 genes respectively, showed differential gene expression between males and females (p = 0.001). 42 genes were differentially expressed both in Mhm*BN and Mhm rats, 52 in Mhm*BN and US, 154 in Mhm and US, and 39 in all three groups.

### Genes that were differentially expressed by gender in all tested groups exhibited the same tendency of gender-dependent expression change among groups

**Figure 2:**
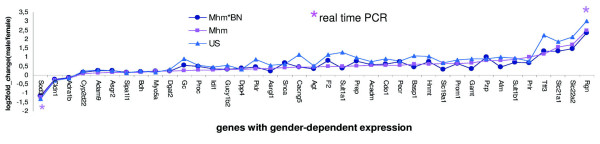
**The same tendency of gender-dependent expression change in all examined groups**. Shown are microarray data for genes which were differentially expressed by gender in all three rat groups. For the two genes (Rgn and Sod3) with strongest expression changes between genders, the microarray data were confirmed by real-time PCR in Mhm rats.

The number of gender-dependently expressed genes was highly influenced by genetic background. For the 39 genes that were differentially expressed by gender in all examined groups, the tendency to up-or down-regulation of the gender-dependent expression change (expressed as log2 fold-change (male/female) in figure 2), was the same among all groups. This means that if one gene was more strongly expressed in one gender in one rat group, it was also more strongly expressed in the same gender in the other two groups, although the dimension of gender-dependent expression change could vary from group to group.

### Real time PCR confirmed gender-dependent gene expression of Rgn and Sod3

Real time PCR was performed to examine the gender-dependent expression of Sod3 and Rgn, the two genes with the most pronounced gender-dependent expression on microarrays (figure 2). Real-time PCR confirmed not only the gender regulated expression of both genes but also the dimension of the gender-dependent expression changes measured by real time PCR was very much similar to that measured with microarrays.

### Gender-dependent gene expression suggests higher metabolic and transport activities, enhanced stimulation of cell proliferation, elevated oxidative stress and altered vascular biology in males compared with females

According to the biological processes in which they participate, genes differentially expressed by gender are clustered in five functional groups: 1) metabolic enzymes and transport proteins; 2) cell proliferation; 3) oxidative stress; 4) vascular biology and 5) signal transduction. Genes that were differentially expressed by gender and the biological processes in which they are involved are summarized in Table 1. The functional annotation of genes was mainly according to Entrez Gene [10].

**Table 1:** Genes differentially expressed by gender and the relevant functional groups.

symbol	full name	biological processes	fold_change(male/female)		
			
			Mhm*BN	Mhm	US
*Adam9*	a disintegrin and metalloproteinase domain 9	metabolism	1.2	1.1	1.2
*Bdh*	3-hydroxybutyrate dehydrogenase	metabolism	1.1	1.1	1.2
*Cdo1*	cysteine dioxygenase 1	metabolism	1.5	1.5	1.9
*Cyp2d22*	cytochrome P450, family 2, subfamily d, polypeptide 22	metabolism	1.1	1.1	1.2
*Dgat2*	diacylglycerol O-acyltransferase homolog 2	metabolism	1.2	1.2	1.3
*Gamt*	guanidinoacetate methyltransferase	metabolism	1.3	1.6	1.9
*Hnmt*	histamine N-methyltransferase	metabolism	1.7	1.5	2.1
*Idi1*	isopentenyl-diphosphate delta isomerase 1	metabolism	1.3	1.2	1.4
*Pecr*	peroxisomal trans-2-enoyl-CoA reductase	metabolism	1.7	1.5	1.7
*Pklr*	pyruvate kinase, liver and red blood cell	metabolism	1.4	1.3	1.9
*Sult1a1*	sulfotransferase family 1A, phenol-preferring, member 1	metabolism	1.3	1.4	2.4
*Sult1b1*	sulfotransferase family 1B, member 1	metabolism	1.6	1.9	1.9
*Prep*	prolyl endopeptidase	metabolism; vascular biology	1.7	1.4	2
*Gc*	group specific component	metabolism; transport	1.5	1.2	1.9
*Pzp*	pregnancy-zone protein	metabolism; signal transduction	2	1.8	1.9
*Dpp4*	dipeptidylpeptidase 4	metabolism; signal transduction	1.3	1.2	1.3
*Cacng5*	calcium channel, voltage-dependent, gamma subunit 5	transport	1.4	1.4	2.2
*Prom1*	prominin 1	transport	1.6	1.6	1.8
*Slc21a1*	solute carrier organic anion transporter family, member 1a1	transport	2.5	2.9	3.7
*Slc22a2*	solute carrier family 22 (organic cation transporter), member 2	transport	2.8	3.2	4.3
*Myo5a*	myosin VA	transport	1.2	1.2	1.1
*Rgn*	regucalcin	transport; signal transduction	5.1	5.6	8
*Basp1*	brain abundant, membrane attached signal protein 1	proliferation	1.4	1.5	2.1
*Tff3*	trefoil factor 3	proliferation	2.5	2.3	4.7
*Sod3*	superoxide dismutase 3, extracellular	oxidative stress	0.5	0.4	0.4
*Acadm*	acyl-Coenzyme A dehydrogenase, C-4 to C-12 straight chain	oxidative stress; metabolism	1.5	1.5	1.7
*Snca*	synuclein, alpha	oxidative stress; signal transduction	1.6	1.3	1.5
*Afm*	afamin	oxidative stress; transport	1.4	1.8	2
*Slc19a1*	solute carrier family 19, member 1	oxidative stress; transport	1.2	1.6	1.6
*Agt*	angiotensinogen (serpin peptidase inhibitor, clade A, member 8)	vascular biology	1.3	1.4	1.4
*F2*	coagulation factor II	vascular biology	1.8	1.4	2.2
*Proc*	protein C	vascular biology	1.4	1.2	1.5
*Gucy1b2*	guanylate cyclase 1, soluble, beta2	vascular biology; signal transduction	1.3	1.2	1.5
*Adra1b*	adrenergic receptor, alpha 1b	vascular biology; signal transduction	0.9	0.9	0.9
*Sipa1l1*	signal-induced proliferation-associated 1 like 1	signal tranduction	1.1	1.1	1.1
*Cldn1*	claudin 1	signal transduction	0.8	0.8	0.8
*Asgr2*	asialoglycoprotein receptor 2	signal transduction	1.2	1.1	1.2
*Asrgl1*	asparaginase like 1	signal transduction	1.2	1.3	1.5
*Prlr*	prolactin receptor	signal transduction	1.6	2	1.7

The majority of the genes expressed differentially in the two sexes code for metabolic enzymes or transport proteins. Out of 39 gender-dependently expressed genes, 15 code for metabolic enzymes and seven may function as transporters. Noticeably, all these genes were more strongly expressed in males. The same was true for two proliferation-relevant genes. Oxidative stress might be elevated through, among others, the strong under-expression of the antioxidant Sod3 in males compared with females. The differential expression of six genes regulating vascular tone and blood coagulation indicates a difference in vascular biology between the genders. Additionally, several genes involved in signal transduction were differentially expressed by gender. In summary, the gender-dependent gene expression suggests higher metabolic and transport activities, enhanced stimulation for cell proliferation, elevated oxidative stress and altered vascular biology in males compared with females.

### Increased superoxide anion in males, particularly during early puberty, is partially caused by reduced anti-oxidation via Sod3

**Figure 3:**
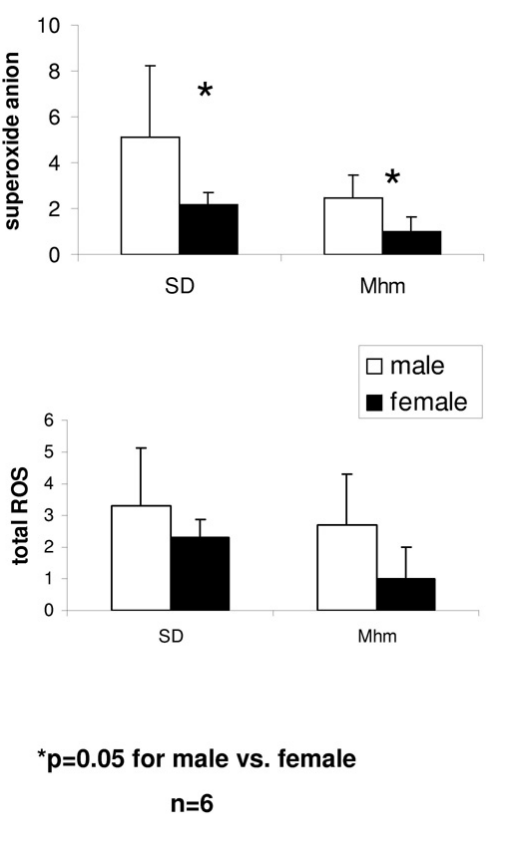
**Elevated superoxide anion in males at early puberty compared with females**. ROS was examined in the glomeruli of 36 day-old SD and Mhm rats. Superoxide anion was significantly higher in males compared with females. Males had the tendency to produce more total ROS, but the gender difference was not statistically significant.

Luminol- and lucigenin-derived chemiluminescence was used to detect total ROS and superoxide anion, respectively. In glomeruli of 36 day-old SD and Mhm rats, superoxide anion was almost 2.5 fold significantly higher in males compared with females (p = 0.05). In both rat groups, the mean of the total ROS was two to three times higher in males compared with females, yet the gender difference was not statistically significant (figure 3).

**Figure 4:**
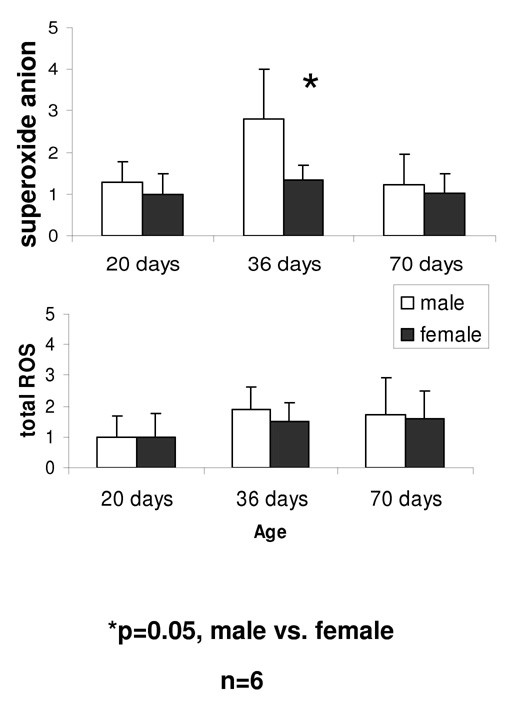
**Development of the gender difference in ROS with age**. Total ROS rose at early puberty and remained at the elevated level during late puberty/early adulthood, without significant differences between genders. At pre-puberty and at late puberty/early adulthood, superoxide anion did not differ significantly between genders. Superoxide anion peaked at early puberty, more abruptly in males than in females (p = 0.05), and dropped to pre-puberty levels as late puberty/early adulthood approached.

To examine the development of the gender difference in ROS with age, ROS was measured in SD rats at the age of 20, 36 and 70 days, corresponding to pre-puberty, early puberty and late puberty/early adulthood, respectively (figure 4). At pre-puberty and late puberty/early adulthood, superoxide anion did not differ significantly between genders. Its level peaked at early puberty, more strongly in males compared with females, and dropped to pre-puberty levels as adulthood approached. Statistically significant elevation of superoxide anion in males compared with females was detected only in rats at early puberty. Total ROS rose at early puberty and remained at the elevated level during late puberty/early adulthood, without significant differences between males and females.

**Figure 5:**
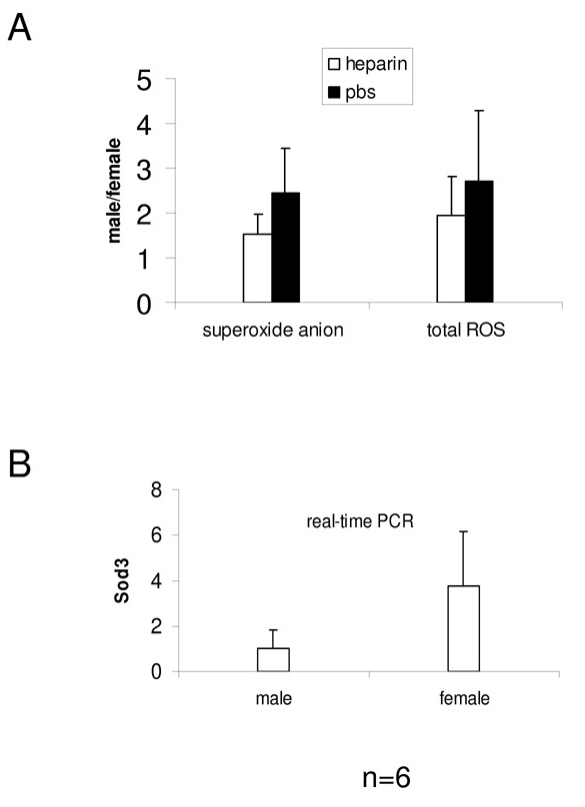
**Reduced anti-oxidation via Sod3 in males compared with females**. A: Gender difference in ROS was examined in glomeruli of 36 day-old Mhm rats, perfused with heparin in PBS or with PBS alone. The elevation of ROS in males is shown on the y-axis (expressed as the ratio of male vs. female). In PBS-perfused kidneys, the elevation of superoxide anion in males was statistically significant. This statistical significance diminished when kidneys were flushed with heparin. Also the elevation of total ROS in males attenuated (the ratio of male vs. female became smaller). B: Real-time PCR confirmed significant under-expression of Sod3 in males compared with females (p < 0.01).

Sod3 is the main anti-oxidant in extracellular spaces. Real time PCR has confirmed the strong down-regulation of Sod3 in males compared to females. In order to assess the contribution of Sod3 to the gender-related elevation of ROS in males, ROS was also measured in rats perfused with PBS containing heparin. The native form of Sod3 exhibits a high heparin-binding capacity [11], therefore, perfusion with heparin can be used to flush Sod3 from organs. The elevation of ROS in males over that in females decreased when kidneys were flushed with heparin (figure 5). The attenuation of the elevation of ROS in males upon the loss of Sod3 function indicates reduced anti-oxidation through Sod3 in males compared with females.

## Discussion

Accelerated deterioration and aging of kidneys in males compared with females has often been documented. To understand the underlying molecular mechanisms, we analysed gender-dependent gene expression in kidneys of rats using microarrays. According to the biological processes in which they participate, genes differentially expressed by gender are clustered in five functional groups (table 1). This result revealed gender differences for genes in more functional groups than formerly published [12], where mainly transport and drug metabolism genes were found to be differentially expressed by gender in kidneys of adult mice.

Sod3, the most efficient antioxidant in extracellular spaces, was significantly under-expressed in males. This under-expression of antioxidant suggests elevated oxidative stress in males compared with females. Total ROS and superoxide anion, a ROS molecule that can diffuse into extracellular spaces, were then detected in isolated glomeruli of 36 day-old Mhm rats. The ROS data confirmed our expectation for the increased oxidative stress in males compared with females. At early puberty, two to three times higher level of superoxide anion was detected in males compared with females (p = 0.05). The elevation of superoxide anion in males in early puberty seems to be a general feature, since it was recorded in both inbred Mhm rats and the reference group SD rats. Superoxide anion can cross cell membranes and diffuse into extracellular spaces, therefore the elevation of superoxide anion detected in glomeruli in males reflects tubulointerstitial hypoxia in kidneys in males. Consistent with the 'free-radical theory of aging' formulated by Harman [13], chronic tubulointerstitial hypoxia is considered the common pathway for renal failure [14]. Chemiluminescence measurements confirmed elevated superoxide anion levels in males compared with females at early puberty. This suggests that early puberty compromises kidney function in males through elevated oxidative stress.

This ROS-data agrees well with the telomere data published by Cherif [2]. ROS shortens telomeres, and telomere length is inversely correlated with biological age. We recorded significantly stronger superoxide anion in kidneys of 36-day old male rats compared with females of the same age. Between the ages of 21 days and three months, Cherif reported significant telomere shortening in kidneys of male rats, but not in kidneys of female rats.

The elevated oxidative stress of males in early puberty compared with females might be caused partially by over-production of oxidative stress factors through over-activities in growth, metabolic and transport, and partially by compromised antioxidation through Sod3. Upon the loss of Sod3 function by flushing the kidney with highly concentrated heparin, the elevation of ROS in males attenuated, but did not diminish completely.

**Figure 6:**
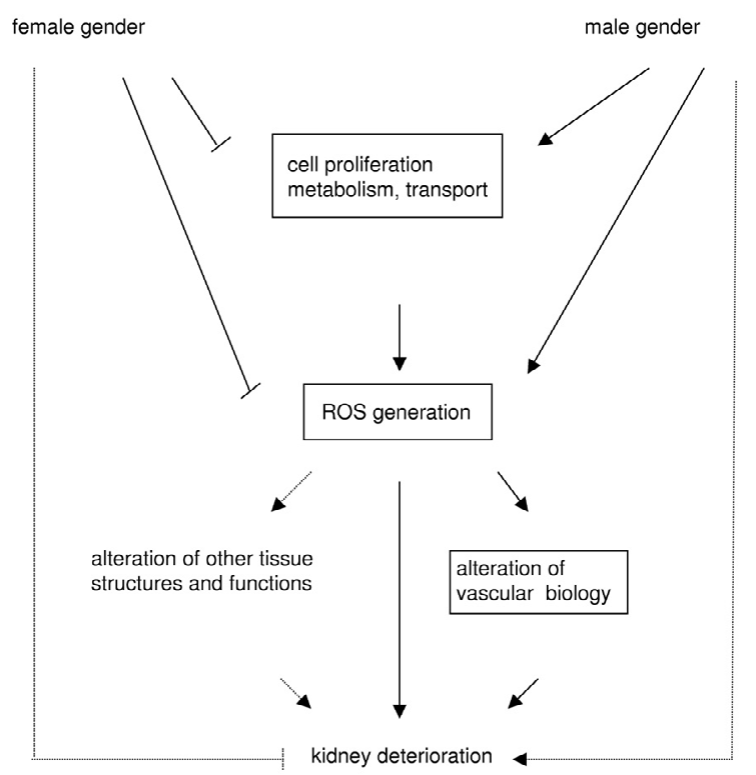
**The proposed mechanism underlying the gender difference in kidney function at early puberty**. Male gender stimulates growth and proliferation, enhances metabolic and transport activities and therefore elevate oxidative stress. Elevated oxidative stress in form of chronic tubulointerstitial hypoxia causes renal failure by altering tissue structures and function such as the vascular biology by elevating blood pressure, damaging vessels and enhancing blood coagulation.

Given that our genomewide transcriptional profiling using microarrays has detected most of the differentially expressed genes in kidneys between genders at early puberty (which is the onset of most gender differences in kindeny function), a network of these differentially expressed genes (or their functional groups) should have the potential to explain the mechanism underlying the gender difference in kidney function. Gender-dependent gene expression implies over-activities in proliferation, metabolism and transport, elevated oxidative stress and altered vascular biology in males compared with females at early puberty. Attemptting to explain the gender-dependent rate of kidney deterioration at early puberty mainly based on the observed transcriptional profile, we suggest that the following possible mechanism might be plausible (figure 6): male gender stimulates growth and proliferation, enhances metabolic and transport activities and elevates oxidative stress, by elevating ROS production and reducing antioxidation through Sod3 [15]. Elevated oxidative stress in the form of tubulointerstitial hypoxia prompts kidney deterioration by altering the renal structures or functions such as vascular biology, including blood pressure elevation, blood vessel damage, and enhanced blood coagulation.

Increased oxidative stress in males compared with females was recorded at early puberty, but neither at pre-puberty nor at late puberty/early adulthood. This implies a particularly strong negative impact of early puberty on kidney in males. Elevated deterioration of kidney function in adult males is most probably not directly caused by increased oxidative stress, but by a longer-lasting negative impact of early puberty. Increased oxidative stress on males in early puberty may cause structural or functional changes that compromise the ability of the animals to endure stress later in life.

## Conclusion

In rats at early puberty, the gene expression profile suggests higher metabolic and transport activities, enhanced cell proliferation, elevated oxidative stress and altered vascular biology in males compared with females. Chemiluminescence measurement confirmed increased superoxide anion and compromised Sod3 antioxidation in males compared with females, particularly at early puberty. These data suggest that early puberty, with gender-related elevation in oxidative stress in males, is a key compromising factor on kidney structure and function in males.

## Methods

### Rat strains

Sprague Dawley (SD) rats and Han:SPRD [16], a rat model for autosomal dominant polycystic kidney disease (PKD) were investigated. Inbred rats originating from the Han:SPRD rat were obtained either from a breeding center in the USA (Mayo Clinic College of Medicine, Rochester, MN, USA hereafter referred to as US) or maintained in our animal care facility (Mannheim, Germany (registered as PKD/Mhm, hereafter designated Mhm). Mhm*BN is a backcross generated of Mhm and Brown Norway rats. In all three rat groups, strongly elevated PKD progression in males compared with females was recorded.

### Gene expression profiling with microarrays

Gender-dependent gene expression was examined in kidneys of 36 day-old Mhm, US and Mhm*BN rats. RNA isolation, cDNA and cRNA synthesis, and hybridization to arrays of type Rae230A from Affymetrix (Santa Clara, CA, USA) were performed according to the recommendations of the manufacturer. RNA from three rats was pooled for each array. Three arrays were used for each gender and group combination. A total of 18 arrays were hybridized. Microarray data were submitted to NCBI GEO [17], sample number [GSE3778].

Microarray data was analysed based on ANOVA using a commercial software package called Micro Array Solution, version 1.0, from SAS (SAS Institute, Cary, NC, USA). Standard settings were used, except the following specifications: log-linear mixed models [18] were fitted for values of perfect-matches, with gender and rat group considered to be constant and the array-id random. Custom CDF [19]with Unigene based gene/transcript definitions different from the original Affymetrix probe set definitions was used to annotate the arrays.

### Verification of microarray data with real-time PCR

Gene expression of Rgn and Sod3 was verified with real-time PCR in Mhm rats, using TaqMan assays predesigned by Applied Biosystems (Forster City, CA, USA).

### Chemiluminescence measurements of ROS

The detection of ROS was carried out as described by Scheuer [20], with slight modifications. Animals werer deeply anesthesized and perfused with 20 ml ice cold PBS. The left kidney was minced and the fraction between 40 μm and 100 μm containing mainly glomeruli was collected with the sieving method. Luminol chemiluminescence was taken as a general detector of ROS (total ROS) and lucigenin chemiluminescence was used to detect superoxide anion diffused into extracellular spaces. ROS was measured at 37°C using a Fluoroskan Ascent (Thermo Labsystems, Waltham, MA, USA), with 500–2000 glomeruli, 5 μM phorbol myristate acetate (PMA, Sigma, St. Louis, MO, USA) and 260 μM luminol (Sigma, St. Louis, MO, USA) or 750 μM lucigenin (Sigma, St. Louis, MO, USA) in 50 μl modified DMEM buffer (Biochrom). Chemiluminescence was recorded for 40 minutes. The ROS capacity was standardized to the amount of total proteins in each assay. The quantification of protein was performed using a BCA Protein Assay Reagent Kit (Pierce, Rockford, IL, USA) on a SLT Rainbow Thermo Elisa Reader (SLT Labinstruments Deutschland GmbH, Crailsheim, Germany).

Elevated oxidative stress in males was first investigated in 36 day-old Mhm rats. To check the universal validity of this gender-dependent elevation of oxidative stress and its development with age, ROS was examined in SD rats at the age of 20, 36 and 70 days. To examine the gender-dependent antioxidation through Sod3, ROS was also measured in rat kidneys perfused with 20 ml PBS including 25,000 units of heparin to flush out Sod3.

Six rats of each group were taken for the ROS examination. The comparison between genders was performed with ANOVA (SAS version 9.1, SAS Institute, Cary, NC, USA). Values were expressed as means ± standard deviation.

## Authors' contributions

LL conducted the microarray analyses and drafted the manuscript. NG participated in the design of the study. LL, SB and PJ participated in ROS measurements. All authors read and approved the final manuscript.
